# Magnetic Resonance Imaging Findings in Infants with Severe Traumatic Brain Injury and Associations with Abusive Head Trauma

**DOI:** 10.3390/children9071092

**Published:** 2022-07-21

**Authors:** Nikki Miller Ferguson, Susan Rebsamen, Aaron S. Field, Jose M. Guerrero, Bedda L. Rosario, Aimee T. Broman, Paul J. Rathouz, Michael J. Bell, Andrew L. Alexander, Peter A. Ferrazzano

**Affiliations:** 1 Department of Pediatrics, Virginia Commonwealth University, Richmond, VA 23298, USA; nikki.millerferguson@vcuhealth.org; 2 Department of Radiology, University of Wisconsin, Madison, WI 53792, USA; srebsamen@uwhealth.org (S.R.); afield@uwhealth.org (A.S.F.); 3 Waisman Center, University of Wisconsin, Madison, WI 53705, USA; jguerrerogon@wisc.edu (J.M.G.); andy.alexander@wisc.edu (A.L.A.); 4 Department of Medical Physics, University of Wisconsin, Madison, WI 53705, USA; 5 Waisman Brain Imaging Laboratory, University of Wisconsin, Madison, WI 53705, USA; 6 Department of Epidemiology, School of Medicine, University of Pittsburgh, Pittsburgh, PA 15213, USA; brosario@gmail.com; 7 Department of Biostatistics and Medical Informatics, University of Wisconsin, Madison, WI 53705, USA; abroman@biostat.wisc.edu; 8 Department of Population Health, University of Texas at Austin Dell Medical School, Austin, TX 78712, USA; paul.rathouz@austin.utexas.edu; 9 Department of Pediatrics, Children’s National Medical Center, Washington, DC 20010, USA; mbell@childrensnational.org; 10 Department of Psychiatry, University of Wisconsin, Madison, WI 53705, USA; 11 Department of Pediatrics, University of Wisconsin, Madison, WI 53705, USA

**Keywords:** traumatic brain injury, pediatrics, magnetic resonance imaging, abusive head trauma

## Abstract

Young children with severe traumatic brain injury (TBI) have frequently been excluded from studies due to age and/or mechanism of injury. Magnetic resonance imaging (MRI) is now frequently being utilized to detect parenchymal injuries and early cerebral edema. We sought to assess MRI findings in infants with severe TBI, and to determine the association between specific MRI findings and mechanisms of injury, including abusive head trauma (AHT). MRI scans performed within the first 30 days after injury were collected and coded according to NIH/NINDS Common Data Elements (CDEs) for Neuroimaging in subjects age < 2 years old with severe TBI enrolled in the Approaches and Decisions in Acute Pediatric Traumatic Brain Injury Trial. Demographics and injury characteristics were analyzed. A total of 81 children were included from ADAPT sites with MRI scans. Median age was 0.77 years and 57% were male. Most common MRI finding was ischemia, present in 57/81 subjects (70%), in a median of 7 brain regions per subject. Contusion 46/81 (57%) and diffuse axonal injury (DAI) 36/81 (44.4%) subjects followed. Children were dichotomized based on likelihood of AHT with 43/81 subjects classified as AHT. Ischemia was found to be significantly associated with AHT (*p* = 0.001) and “inflicted” injury mechanism (*p* = 0.0003). In conclusion, the most common intracerebral injury seen on MRI of infants with severe TBI was ischemia, followed by contusion and DAI. Ischemia was associated with AHT, and ischemia affecting > 4 brain regions was predictive of AHT.

## 1. Introduction

Traumatic brain injury (TBI) remains a leading cause of death and disability in young children, with an annual incidence of TBI-related emergency department visits and hospitalization for children < 4 years old of ~1660 per 100,000 in the United States [1]. Infants with severe TBI represent a unique population due to differences from older children in biomechanical properties of the brain and skull, and in the most common causes of injury [2,3]. However, the impact these differences have on subsequent brain injury remain poorly understood.

One of the most distinctive aspects of severe TBI in infants is the high incidence of abusive head trauma (AHT) in this population. Abusive head trauma (AHT) is the leading cause of fatal head injuries in this age group, with an annual incidence of 33 to 38 per 100,000 infants [4,5] and high rates of mortality and long-term disability [6,7,8,9]. Diagnosing AHT is a clinical challenge that requires a combination of physical exam, detailed history and diagnostic studies including neuroimaging [10]. In recent years, magnetic resonance imaging (MRI) has been found to be a useful and sensitive modality for detecting parenchymal injuries, including contusions, diffuse axonal injury (DAI), hypoxic-ischemic injury (HII), and early cerebral edema in TBI. Early studies suggested that DAI was the predominant injury type in AHT; however, more recent studies using diffusion and susceptibility-weighted MRI sequences have indicated a lower incidence of DAI, and instead suggest an association between HII and AHT [11,12,13,14]. MRI is now a recommended diagnostic tool for AHT when used in conjunction with history, physical exam, and laboratory findings [10].

The objective of this study was to characterize MRI findings in infants with severe TBI, and to determine the association between specific MRI findings and mechanisms of injury, including AHT. This study was conducted in collaboration with the Acute Decisions and Approaches in Pediatric TBI (ADAPT) trial, which enrolled children with severe TBI (Glasgow Coma Scale (GCS) < 9) from any cause [15,16,17]. The ADAPT trial is unique compared to many previous studies in that it included children with proven or suspected AHT, whereas many pediatric TBI studies have excluded this mechanism of injury [15,18,19,20]. In the present study, MRI scans performed as part of the clinical care of children with severe TBI within the first 30 days of injury were collected from participating ADAPT sites. MRI findings and associations with mechanism of injury are presented in this large cohort of infants with severe TBI.

## 2. Materials and Methods

### 2.1. Participants

The ADAPT trial was a prospective observational study that enrolled 1000 children with severe TBI defined as a post-resuscitation GCS < 9. Twenty-four ADAPT trial clinical sites participated in the MRI Sub-study. Participating sites submitted the first brain MRI scan acquired within 30 days of injury in all ADAPT subjects enrolled at their site who had an MRI performed as part of standard clinical care. The MR scanning protocols were determined by the clinical standard practice in use at each site. The current study focused on the 81 MRI scans collected in children less than 2 years of age. This study was approved by the institutional review board at the University of Pittsburgh (ADAPT Coordinating Center), University of Wisconsin (MRI Sub-study Coordinating Center), and all participating sites. All sites were permitted to perform data collection before informed consent was obtained. Written informed consent was obtained from parents/guardians for long-term outcome assessments that are not a part of the study presented here. Informed consent was not required for data collection at all clinical sites. The cohort therefore represents consecutive children who met the inclusion criteria at participating sites. We followed the STrengthening the Reporting of OBservational studies in Epidemiology (STROBE) statement guidelines in the preparation of this manuscript.

### 2.2. MRI Analysis

MRI scans were read by one of two board certified neuroradiologists, each with >20 years of clinical experience. MRI findings were coded according to the NIH/NINDS Common Data Elements (CDEs) for Neuroimaging as described by Haacke et al. [21]. For measurement of contusion volume, the greatest extent was measured in 3 orthogonal planes and an idealized ellipsoid volume was calculated (ABC*pi/6). For quantification of diffuse axonal injury, microhemorrhages in white matter were counted in each brain region up to a total of 10 lesions, and regions with >10 lesions received a score of 11. Ischemia was defined as evidence of tissue injury on diffusion-weighted imaging (DWI) consistent with a deficit between substrate demand and delivery, scored as present/absent in each brain region and quantified as the number of brain regions affected by that lesion type. The brain regions assessed for each lesion can be found in Supplemental Table S1. Midline shift was determined by measuring the displacement from midline of any anatomical landmark normally located at the midline (e.g., septum pellucidum). Intraventricular hemorrhage was quantified as the number of ventricles with hemorrhage. The measures included in this analysis are restricted to intracerebral findings due to the high prevalence of device placement (all subjects had intracranial pressure (ICP) monitor or external ventricular drain (EVD)) and craniectomy (~40% of subjects), making assessment of extra-axial fluid/blood unreliable.

### 2.3. Injury Characteristics

The following injury variables were characterized for each subject by review of the medical record: the cause of injury (categorized as motor vehicle, fall, inflicted, or other), the mechanism of injury (categorized as acceleration/deceleration, impact, crush, fall, gunshot, or other), whether the injury was a closed vs. open head injury type, and whether the injury was due to AHT. AHT was stratified based on the certainty of the diagnosis determined at each site as previously described [17,18]. “Definite” indicates that a medical diagnosis of child abuse was made by a healthcare professional at the clinical site; “probable” indicates that the clinical care team believed the child was a victim of abuse but the diagnosis was not confirmed; “possible” indicates that the possibility of child abuse was considered in the differential diagnosis but was neither confirmed nor excluded, and “no abuse” indicates that no documentation exists in the medical record that abuse was considered. For this analysis, we combined subjects with “Probable AHT” and “Definite AHT” into an “Abuse” group, and combined the “No AHT” and “Possible AHT” subjects into the “No Abuse” group. We chose to include “Possible AHT” subjects in the No Abuse group as a more conservative classification, limiting the subjects in the “Abuse” group to those with confirmed or very high-suspicion for abuse. We performed a sensitivity analysis by testing for associations with abuse after excluding the “Possible Abuse” subjects.

### 2.4. Statistical Approach

Demographics and injury characteristics were summarized using descriptive statistics. Imaging findings were described for the whole brain and then subsequent analyses were restricted to the five most common imaging findings (DAI, contusion, ischemia, midline shift, and intraventricular hemorrhage). Associations between imaging findings and the demographic and injury variables were modeled using semiparametric generalized linear models with a log link [22]. This model permits valid likelihood ratio tests (LRT) and confidence intervals that are typically smaller in width and hence more powerful than Poisson regression. Coefficient estimates are interpreted as relative means, as with log-linear Poisson regression. For injury variables with multiple categories (i.e., mechanism), the LRT tests whether the imaging measures are associated with that overall injury variable, rather than testing the association with each category within that variable. Each injury outcome was analyzed in a univariate model with age, sex, GCS, abuse (yes/no), injury cause (reference category: motor vehicle), injury type (closed or open), and injury mechanism (reference: Acceleration/Deceleration). We adjusted the *p*-values across all univariate regression models to control the family-wise error rate using the Hochberg method [23], and considered significant association with *p* ≤ 0.05. A Receiver Operating Characteristic (ROC) curve was plotted for ischemia predicting abuse, with the true-positive rate plotted against the false-positive rate. The optimal threshold for classifying the outcome (abuse yes/no) was determined as the value where sensitivity and specificity are maximized, and the absolute difference between sensitivity and specificity is minimized. The area under the ROC curve (AUC) indicates how well the measure classifies the outcome of importance, with AUC = 0.5 having no separation capacity, and AUC = 1 having perfect separation capacity.

## 3. Results

Demographics and injury characteristics are presented in Table 1 (online) for the 81 children included in this analysis. There was a greater proportion of males (57%), and the median age was 0.77 years (IQR 0.30, 1.37). Over half of the subjects were classified as AHT. GCS scores ranged from 3 to 8, and the most common mechanism of injury was impact.

**Figure 1:**
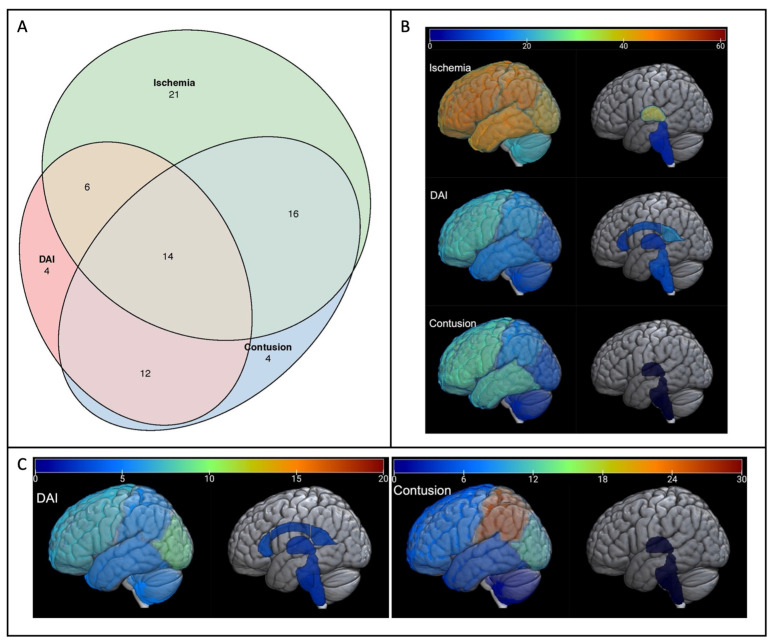
Patterns of injury in infants with severe TBI. The three most common intracerebral lesion types are shown: Ischemia, Contusion, and Diffuse Axonal Injury (DAI). (**A**) Venn diagram of MRI lesions. Number of subjects with each lesion type and co-occurrence of lesions is shown. (**B**) Lesion Frequency Maps. Heat maps demonstrating number of subjects with lesion in each brain region are shown. For cerebral lobes, this represents number of subjects with lesion in either hemisphere. (**C**) Lesion Quantification Maps. Heat maps demonstrating median DAI lesion count and contusion volume (cc) in each brain region are shown. For cerebral lobes this represents the total of both hemispheres. Regional ischemia was not quantified within brain regions.

Intracerebral MRI findings are summarized in Table 2. Ischemia was the most common injury type, present in 57/81 subjects (70%), and was found in a median of seven brain regions per subject. Contusion was observed in 46/81 (57%) subjects with a median total lesion volume of 21 cc. The 36/81 (44.4%) subjects with DAI had a median of 12.5 microhemorrhages. Intraventricular hemorrhage was observed in 33% of subjects, and midline shift in 24.7% with a median shift of 3 mm (IQR 2, 6.2). The remaining MRI measures were found in <10% of subjects. Figure 1a shows a graphic representation of how the top three most common injuries (ischemia, contusion, and DAI) co-occur in subjects. Over half of the subjects had at least two of these injury types, with 14/81 being afflicted with all three injury types. In 25% of infants, ischemia was present without either DAI or contusion. The regional distributions of these three most common injuries are shown in Figure 1b,c. As seen in Figure 1b, the frontal lobe was the most commonly affected region for each lesion type, while brainstem injury was uncommon. In contrast, the burden of injury (Figure 1c) was highest in the parietal lobe for contusion and in the occipital lobe for DAI. Exact values for frequency and quantification, along with interquartile ranges, can be found in Supplemental Table S1.

**Figure 2:**
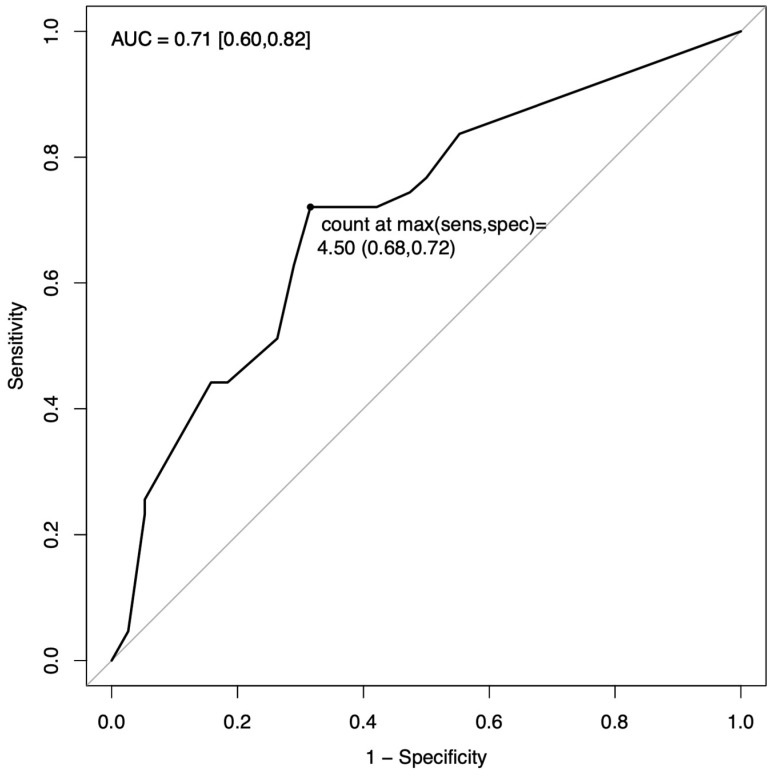
Receiver Operating Characteristic (ROC) curve for ischemia predicting abuse. Area under the curve (AUC) with 95% CI is shown for the number of brain regions with ischemia. The maximum sensitivity and specificity threshold occurred at 4.5 ischemic regions.

Next, whole brain quantification values for DAI, contusion, ischemia, midline shift, and IVH were used to determine univariate associations with demographic and cause-of-injury variables. Results for the three most common intracerebral lesion types (ischemia, contusion, and DAI) are shown in Table 3. No association was found between imaging findings and age, sex or GCS. Ischemia was found to be significantly associated with AHT (*p* = 0.001) and “inflicted” injury cause (*p* = 0.0003). IVH was more significantly associated with motor vehicle accidents (*p* = 0.001, Supplemental Table S2). These associations remained significant after Hochberg adjustment for multiple comparisons. In a sensitivity analysis, the association between ischemia and abuse remained significant after excluding the subjects with “Possible Abuse” (Supplemental Table S3). There were no significant associations found with DAI, contusion, or midline shift. Figure 2 shows the ROC curve for ischemic injury burden (number of brain regions affected) predicting AHT. The maximum sensitivity and specificity threshold was reached at >4 regions affected (sensitivity = 0.72, specificity = 0.68). Area under this curve (AUC) is 0.71, with a 95% confidence interval of 0.60–0.82.

## 4. Discussion

Our study represents the largest prospectively collected cohort of severe TBI in children < 2 y who had early (<30 d from injury) MR imaging as part of their clinical workup. First, we described the injury patterns found on MRI scans in infants with severe TBI. The most striking was the high incidence of ischemia; many subjects demonstrated ischemia without contusion or DAI, and when ischemia was present it was generally widespread, affecting a median of seven brain regions. The regional distribution of ischemia was fairly uniform across the cerebral hemispheres, while brainstem ischemia was rare. DAI was most common in the frontal lobes and splenium of the corpus callosum, while the greatest lesion burden was found in the occipital lobes. Interestingly, regional differences in frequency and severity of white matter injury reflect the developmental trajectory of myelination, which proceeds in a caudo-cephalad and posterior-anterior direction. Myelination occurs early in the genu of the corpus callosum and the sub-occipital white matter, [24,25] regions where we found high incidence and burden of micro-hemorrhages. Contusion was most common in the fronto-temporal region as is well-known in adults [26,27] with the greatest lesion burden seen in the parietal lobes. Additional study comparing infant injury patterns to those found in older children may help to elucidate the interplay between brain injury, brain development, and other biomechanical properties of the infant brain, skull, face and neck [2].

Next, we related MRI findings with demographics and injury characteristics. We found no association between imaging findings and age or sex. As has been stated in previous reports of the ADAPT trial, this cohort is unique as it includes children with AHT as the mechanism of injury [17,18]. Over half of the infants included in our study were probable or confirmed victims of AHT, providing us the opportunity to examine the association between MRI findings and inflicted/abusive head injury. Consistent with other reports, AHT victims were predominantly males, and were younger (majority < 1 y) than non-AHT subjects [3]. We found that ischemia was the most common intracerebral injury in this cohort of infants with severe TBI, affecting more than two-thirds of subjects. Additionally, ischemia was significantly associated with AHT and an inflicted mechanism of injury, and ischemia in more than four brain regions was predictive of abuse, with an AUC for ischemia of 0.71 (0.60, 0.82) indicating good separation capacity between groups. It was previously thought that DAI was the predominant injury pattern seen in AHT, but more recent studies have suggested that ischemia is the more common parenchymal injury. Large systematic reviews from Piteau and Kemp have described neuroimaging findings associated with AHT; these studies found that subdural hematoma and cerebral hypoxia-ischemia are suggestive of AHT [11,28]. More recently, Orru’ et al. studied diffusion MRI scans in 57 children with a diagnosis of any severity AHT and found evidence of hypoxia-ischemia in 37% of subjects [13]. In agreement with these studies, we found that ischemia was associated with AHT. As our study focused on infants with severe TBI, the incidence of ischemia was higher than that observed in the Orru study. To our knowledge, this is the first effort to establish an ischemia burden that is suggestive of abuse. We determined that >4 ischemic regions is the optimal threshold for classifying subjects into the Abuse vs. No Abuse groups, balancing detection of true-positives while minimizing false-positives (the value where sensitivity and specificity are maximized, and the absolute difference between sensitivity and specificity is minimized). Similarly, Binenbaum et al. found that AHT was associated with retinal hemorrhages and diffuse ischemia on MRI [29]. AHT is a medical diagnosis and relies on a multidisciplinary assessment of all clinical findings and medical history [10,30]. Our findings indicate that in infants with severe TBI and evidence of cerebral ischemia on MRI, AHT should be considered, especially when many brain regions are affected. Given this, early MRI should be part of the diagnostic workup along with extensive history taking, physical exam, laboratory studies, ophthalmologic exam, and other imaging modalities [3,31].

Interestingly, the brainstem was found to have the least amount of ischemic injury whereas over half of all subjects had supratentorial ischemic injuries. Similarly, there was little DAI and no contusion reported in the brainstem of subjects. It has been previously suggested that injury to the brainstem is the cause of apnea and ensuing hypoxic-ischemic injury associated with TBI in young children and infants [3]. Our data would indicate that ischemia is not dependent on brainstem injury, suggesting that delays in care or other injury mechanisms specific to abuse may account for cerebral ischemia in AHT. Children with AHT are at increased risk of secondary injury during the pre-hospital period due to untreated seizure or hypoxia, as caregivers responsible for the injury may be reluctant to seek medical care [7,32]. Indeed, we previously found that compared to non-AHT, children with AHT were significantly more likely to have been transported from home, (60% vs. 33.5%), have apnea (34.3% vs. 12.3%) and have seizures (28.6% vs. 7.7) [18], consistent with a number of other studies which have reported a high rate of seizure and apnea in the AHT population [33,34,35,36]. The increased incidence of secondary injury and resulting cerebral ischemia may in turn account for the high rates of mortality and long term disability in children with AHT [7,32,37,38].

There are some limitations to this study which must be considered when interpreting our findings. The MRI scans were performed as part of clinical care at each site, so the indication for obtaining MRI and scanning protocols were not standardized across sites. MRI scans were obtained up to 30 days post-injury, so may reflect varying stages of the evolution of brain injury, and inherent with the mechanism of AHT, the exact time of injury is often uncertain. The suspicion of abuse may have contributed to the clinical decision to obtain an MRI scan, and the results of the imaging may have informed the clinical diagnosis of abuse and stratification into the abuse group in our study. The infants who had an MRI scan performed may not be representative of all infants with severe TBI, as an MRI may not be obtained in children who recover quickly or who are expected to die. Lastly, as all of these children had undergone instrumentation (ICP monitor and/or EVD) and many had undergone craniectomy, we could not characterize skull fracture or extra-axial hemorrhages, both of which may be associated with AHT.

## 5. Conclusions

In this study we found that the most common intracerebral injury seen on MRI of infants with severe TBI was ischemia, followed by contusion and DAI. Ischemia was associated with AHT, and ischemia affecting >4 brain regions was predictive of AHT. Our findings support that MRI is useful in the evaluation of infants with suspected AHT, along with detailed history and exam. Cerebral ischemia raises the concern of abuse in children presenting with severe TBI, especially with multiple region involvement.

## Figures and Tables

**Table 1:** Demographics and Injury Characteristics.

		Subjects N = 81
Age (years)	0–0.5	30 (37.0%)
	0.51–1.0	18 (22.2%)
	1.1–1.5	14 (17.3%)
	1.51–2.0	19 (23.5%)
Sex	Female	35 (43.2%)
	Male	46 (56.8%)
GCS Total	3–4	29 (35.8%)
	5–6	30 (37.0%)
	7–8	22 (27.2%)
Abuse	No	38 (46.9%)
	Yes	43 (53.1%)
Cause	MVA	14 (17.3%)
	Fall	16 (19.8%)
	Inflicted	45 (55.6%)
	Other Accidental	6 (7.4%)
Type	Closed	76 (93.8%)
	Penetrating/Crush	5 (6.2%)
Mechanism	Accel/Decel	18 (22.2%)
	Impact	45 (55.6%)
	Crush	2 (2.5%)
	Fall	12 (14.8%)
	Gunshot	2 (2.5%)
	Unknown/other	2 (2.5%)

**Table 2:** Prevalence and quantification of MRI findings.

MRI Finding	N (%)	Quantification	Median (IQR)
Ischemia	57 (70.4)	number of brain regions affected	7 (5, 10)
Contusion	46 (56.8)	total lesion volume (cc)	21.0 (5.6, 48.7)
Diffuse Axonal Injury	36 (44.4)	number of microhemorrhages	12.5 (7.5, 46.8)
Intraventricular Hemorrhage	27 (33.3)	number of ventricles affected	2 (1.5, 2)
Supratentorial Midline Shift	20 (24.7)	distance (mm)	3 (2, 6.2)
Cisternal Compression	8 (9.9)	number of cisterns affected	2 (1, 5)
Venous Sinus Injury	5 (6.2)	number of brain regions affected	1 (1, 2)
Intracerebral Hemorrhage	5 (6.2)	total lesion volume (cc)	3.1 (2.0, 25.6)
Brain Atrophy	4 (4.9)	number of brain regions affected	12 (7.2, 16)
Penetrating Injury	4 (4.9)	number of brain regions affected	1.5 (1, 2)
Edema NOS	3 (3.7)	total lesion volume (cc)	4 (3, 8)
Fourth Ventricle Shift	2 (2.5)	distance (mm)	7.5 (6.2, 8.8)
Brainstem Injury NOS	1 (1.2)	number of brain regions affected	1 (1, 1)
Brain Swelling	1 (1.2)	number of brain regions affected	2 (2, 2)

Median quantification is for subjects with that lesion type. NOS indicates findings not captured in other lesion categories.

**Table 3:** Linear Density Ratio univariate models of lesion type with injury characteristics.

		Estimate	Std. Error	t Value	Pr (>|t|)	F Stat	df1	df2	*p*-Value
**Diffuse Axonal Injury**									
Age		0.356	0.350	1.02	0.31	1.03	1	79	0.31
Sex	Male	0.115	0.444	0.26	0.8	0.067	1	79	0.8
GCS		0.0642	0.1325	0.48	0.63	0.232	1	79	0.63
Abuse	Yes	−0.356	0.436	−0.82	0.42	0.665	1	79	0.42
Cause	MVA		*Reference*			2.15	3	77	0.1
	Fall	−0.607	0.566	−1.07	0.287				
	Inflicted	−1.219	0.482	−2.53	0.014				
	Other Accidental	−1.666	1.074	−1.55	0.125				
Type	Closed		*Reference*			0.027	1	79	0.87
	Penetrating/crush	0.143	0.861	0.17	0.87				
Mechanism	Accel/Decel		*Reference*			0.623	5	75	0.68
	Impact	0.229	0.561	0.41	0.68				
	Crush	−15.857	1033.733	−0.02	0.99				
	Fall	0.290	0.718	0.40	0.69				
	Gunshot	0.123	1.471	0.08	0.93				
	Unknown	−1.221	1.896	−0.64	0.52				
**Contusion**									
Age		0.473	0.331	1.43	0.16	1.72	1	79	0.19
Sex	Male	0.443	0.417	1.06	0.29	1.08	1	79	0.3
GCS		−0.229	0.120	−1.92	0.059	3.52	1	79	0.064
Abuse	Yes	0.349	0.415	0.84	0.4	0.69	1	79	0.41
Cause	MVA		*Reference*			0.055	3	77	0.98
	Fall	−0.167	0.686	−0.24	0.81				
	Inflicted	−0.102	0.570	−0.18	0.86				
	Other Accidental	−0.365	0.927	−0.39	0.69				
Type	Closed		*Reference*			0.011	1	79	0.92
	Penetrating/crush	0.090	0.864	0.1	0.92				
Mechanism	Accel/Decel		*Reference*			1.24	5	75	0.3
	Impact	0.849	0.520	1.63	0.11				
	Crush	−2.887	2.100	−1.38	0.17				
	Fall	0.531	0.682	0.78	0.44				
	Gunshot	1.217	1.224	0.99	0.32				
	Unknown	−1.843	1.907	−0.97	0.34				
**Ischemia**									
Age		−0.129	0.165	−0.78	0.44	0.722	1	79	0.4
Sex	Male	−0.0184	0.1969	−0.09	0.93	0.009	1	79	0.93
GCS		−0.0842	0.0581	−1.45	0.15	2.07	1	79	0.15
Abuse	Yes	0.666	0.215	3.10	0.0027	10.8	1	79	0.0015 *
Cause	MVA		*Reference*			6.97	3	77	0.0003 *
	Fall	0.767	0.504	1.52	0.132				
	Inflicted	1.349	0.440	3.06	0.003				
	Other Accidental	0.228	0.730	0.31	0.755				
Type	Closed		*Reference*			6.51	1	79	0.013
	Penetrating/crush	−1.9374	1.0414	−1.86	0.067				
Mechanism	Accel/Decel		*Reference*			2.3	5	75	0.053
	Impact	−0.203	0.202	−1.01	0.32				
	Crush	−1.212	1.130	−1.07	0.29				
	Fall	−0.420	0.328	−1.28	0.20				
	Gunshot	−21.245	11,197.335	0.00	1.00				
	Unknown	−2.599	1.882	−1.38	0.17				

Motor vehicle accident (MVA); Glasgow Coma Scale (GCS); * Hochberg adjusted *p*-value ≤ 0.05.

## Data Availability

Data collected in this study has been made available through the Federal Interagency Traumatic Brain Injury Research (FITBIR) Informatics System.

## Supplementary Materials

The following supporting information can be downloaded at https://www.mdpi.com/article/10.3390/children9071092/s1. Table S1, Lesion Prevalence and Quantification by Injury Type and Brain Region; Table S2, Linear Density Ratio univariate model of midline shift and intraventricular hemorrhage with injury characteristics; Table S3, Linear Density Ratio univariate model of lesion and abuse, excluding subjects with “Possible Abuse”.
