# Supplementary material for: Magnetic Resonance Imaging Findings in Infants with Severe Traumatic Brain Injury and Associations with Abusive Head Trauma
Source: Children (Basel). 2022 Jul 21;9(7):1092. doi: 10.3390/children9071092 (PMC9322188; doi:10.3390/children9071092)
Supplement: Supplementary file 1 [file children-09-01092-s001.zip › children-1786460-supplementary.pdf]

Supplementary files:

Table S1. Lesion Prevalence and Quantification by Injury Type and Brain Region.

Injury	Region	N (%)	Median [IQR]
Diffuse Axonal Injury		total=36	# of lesions
	Corpus Callosum	24 (29.6)	5 [1,12]
	Brainstem	11 (13.6)	2 [1.5,3.5]
	Cerebellum	9 (11.1)	5 [4,22]
	Frontal Lobes	26 (32.1)	7.5 [2,11]
	Parietal Lobes	20 (24.7)	5.5 [2.8,11]
	Temporal Lobes	17 (21)	5 [2,11]
	Occipital Lobes	10 (12.3)	10 [4.5,11]
	Thalamus/Basal Ganglia	9 (11.1)	2 [1,10]
Contusion		total=46	Lesion volume
	Brainstem	0 (0)	0
	Cerebellum	5 (6.2)	0.7 [0.3,1.6]
	Frontal Lobes	29 (35.8)	7.1 [4.3,21.3]
	Parietal Lobes	19 (23.5)	27.1 [6.6,56.6]
	Temporal Lobes	27 (33.3)	4.3 [2,8.6]
	Occipital Lobes	10 (12.3)	13.3 [6.4,26.6]
	Thalamus/Basal Ganglia	0 (0)	0
Ischemia		total=57	# of regions
	Brainstem	4 (4.9)	NA
	Cerebellum	22 (27.2)	2 [2,2]
	Frontal Lobes	51 (63)	2 [1,2]
	Parietal Lobes	50 (61.7)	2 [1,2]
	Temporal Lobes	46 (56.8)	2 [1,2]
	Occipital Lobes	43 (53.1)	2 [1,2]
	Thalamus/Basal Ganglia	40 (49.4)	2 [1,2]

Table S2. Linear Density Ratio univariate model of midline shift and intraventricular hemorrhage with injury characteristics.

		Estimate	Std. Error	t value	Pr(>|t|)	F stat	df1	df2	p-value
**Midline Shift**									
Age		-0.25386	0.41466	-0.61	0.54	0.384	1	79	0.54
Sex	Male	-1.038	0.494	-2.1	0.039	4.38	1	79	0.04
GCS		0.321	0.154	2.09	0.04	4.19	1	79	0.044
Abuse	Yes	0.0415	0.4916	0.08	0.93	0.0071	1	79	0.93
Cause	MVA		*Reference*			1.1	3	77	0.36
	Fall	-1.198	0.783	-1.53	0.130				
	Inflicted	-0.774	0.539	-1.43	0.155				
	Other Accidental	-1.421	1.237	-1.15	0.254				
Type	Closed		*Reference*			2.94	1	79	0.09
	Penetrating/crush	-21.064	15854.060	0.00	1.00				
Mechanism	Accel/Decel		*Reference*			0.862	5	75	0.51
	Impact	-0.566	0.526	-1.08	0.29				
	Crush	-21.495	25067.478	0.00	1.00				
	Fall	-1.083	0.877	-1.23	0.22				

		Estimate	Std. Error	t value	Pr(>|t|)	F stat	df1	df2	p-value
	Gunshot	-21.495	25067.478	0.00	1.00				
	Unknown	-0.138	1.370	-0.10	0.92				
**Intraventricular Hemorrhage**									
Age		-0.253	0.311	-0.81	0.419	0.669	1	79	0.42
Sex	Male	-0.307	0.364	-0.84	0.40	0.708	1	79	0.4
GCS		0.0188	0.1104	0.17	0.865	0.0294	1	79	0.86
Abuse	Yes	-0.8681	0.3799	-2.28	0.025	5.43	1	79	0.022
Cause	MVA		*Reference*			5.95	3	77	0.001*
	Fall	-1.555	0.534	-2.91	0.0047				
	Inflicted	-1.539	0.355	-4.33	4.4e-05				
	Other Accidental	-1.421	0.784	-1.81	0.0734				
Type	Closed		*Reference*			0.249	1	79	0.62
	Penetrating/crush	0.342	0.645	0.53	0.598				
Mechanism	Accel/Decel		*Reference*			0.772	5	75	0.57
	Impact	-4.05e-01	4.10e-01	-0.99	0.33				
	Crush	-3.96e-17	1.02e+00	0.00	1.00				
	Fall	-6.93e-01	6.50e-01	-1.07	0.29				
	Gunshot	4.05e-01	8.07e-01	0.50	0.62				
	Unknown	-2.03e+01	1.82e+04	0.00	1.00				

Motor vehicle accident (MVA); Glasgow Coma Scale (GCS); * Hochberg adjusted p-value ≤ 0.05.

Table S3. Linear Density Ratio univariate model of lesion and abuse, excluding subjects with "Possible Abuse".

		Estimate	Std. Error	t value	Pr(>|t|)	F stat	df1	df2	P-value
Diffuse Axonal Injury	Abuse Yes	-0.821	0.421	-1.95	0.056	3.54	1	61	0.065
Contusion	Abuse Yes	0.0907	0.4903	0.19	0.85	0.034	1	61	0.85
Ischemia	Abuse Yes	1.481	0.415	3.57	0.00071	19.5	1	61	4.2e-05
